# C2H2-type zinc finger protein transcription factor MdZAT1 plays a negative role in anthocyanin biosynthesis in apple

**DOI:** 10.1186/s43897-025-00150-6

**Published:** 2025-05-08

**Authors:** Yanxue Ren, Wenping Huo, Zhongkang Wang, Shasha Liu, Yizhou Chen, Xiaolong Xu, Hongmin Hou, Chaohua Dong, Jihua Xu, Min Chen, Yugang Zhang, Shenghui Jiang

**Affiliations:** 1 College of Horticulture, Engineering Laboratory of Genetic Improvement of Horticultural Crops of Shandong Province, Qingdao Agricultural University, 700 Changcheng Road, Qingdao, Shandong China; 2 Qingdao Key Lab of Genetic Improvement and Breeding of Horticultural Plants, Qingdao Agricultural University, 700 Changcheng Road, Qingdao, China; 3 College of Life Science, Qingdao Agricultural University, 700 Changcheng Road, Qingdao, China; 4 Shenzhen Branch, Guangdong Laboratory for Lingnan Modern Agriculture, Genome Analysis Laboratory of the Ministry of Agriculture, Agricultural Genomics Institute at Shenzhen, Chinese Academy of Agricultural Sciences, 97 Buxin Road, Shenzhen, China

Apple is a most important fruit tree and contains various nutrients and health-promoting compounds. Fruit color is an important factor in terms of consumer preference and determined by anthocyanin content (Winkel-Shirley, 2001; Jiang et al. 2020). Anthocyanin is synthesized on the cytosolic surface of the endoplasmic reticulum (Gomez et al. 2009; Francisco et al. 2013; Jiang et al. 2019). The anthocyanin biosynthetic pathway is conserved across plant species and occurs via the phenylalanine metabolic pathway (Baudry et al. 2004). Structural genes are the genes coding enzymes involved in this pathway, which including the early and late biosynthetic genes. In addition, the transcriptional regulator MBW complex, which contains two TFs MYB, bHLH and a WD40 protein, also effects anthocyanin biosynthesis(Xu et al. 2015).

C2H2 (Cys-2/His-2)-type zinc finger protein TFs are the largest zinc finger family in plant, and are associated with plant growth and development and responding to kinds of stresses(Ciftci-Yilmaz and Mittler 2008; Kim et al. 2011; Yang et al. 2021). In our previous study, a MYB protein, MdMYB114, could activate the expression of *MdANS*, *MdUFGT*, and *MdGST* to positively regulate anthocyanin biosynthesis(Jiang et al. 2021). Further, Y1H assay showed that *MdMYB114* was regulated by a C2H2-type zinc finger protein TF MdZAT1, which probably interacts with the *MdMYB114* promoter to affect anthocyanin accumulation (Table S1). However, the role of MdZAT1 in the anthocyanin biosynthetic pathway remains unclear.

To investigate the function of MdZAT1, an analysis of *MdZAT1* CDS firstly cloned from the fruit peel showed that the gene encoded a protein of 347 amino acids with a calculated molecular mass of 41.64 kDa. We also found there is a predicted C2H2-type zinc finger domain located between 183 amino acids (AA) and 205 AA (Fig. S1A). Further the results of phylogenetic and sequence analyses of MdZAT1 showed that MdZAT1 is closely related to the anthocyanin-related ZAT proteins, so that MdZAT1 may also be associated with stress and anthocyanin accumulation (Fig. S1B and 1A). Moreover, the 35S:*MdZAT1*-GFP was transiently expressed in tobacco leaf cells, he leaves showed diffused fluorescence throughout the cell in the control, while the leaves with the MdZAT1-GFP construct displayed fluorescence only in the nucleus, indicating MdZAT1 localization in the nucleus (Fig. 1B).

**Fig. 1:**
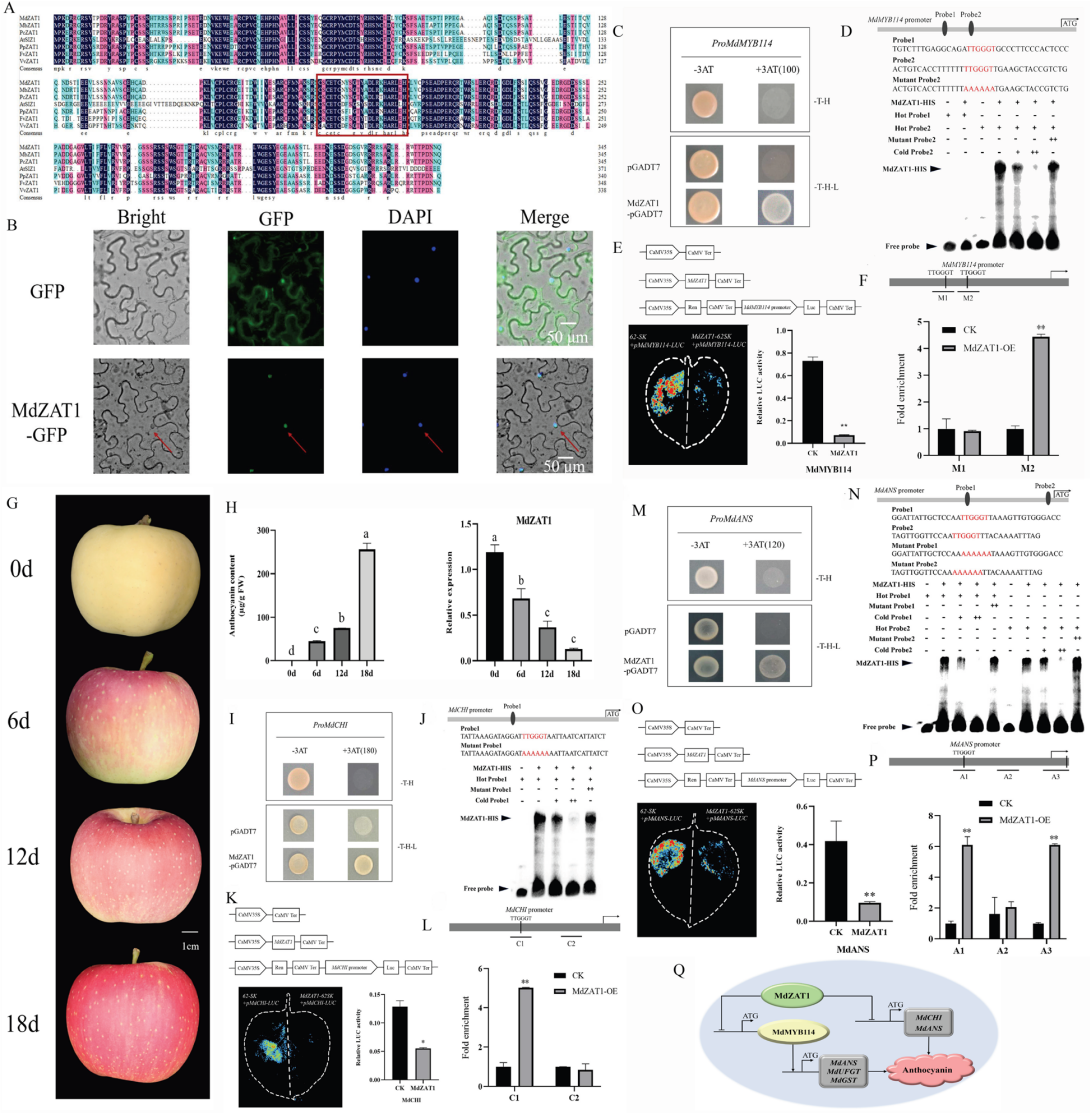
**A** Multiple sequence alignment of homologous amino acid sequences of different C2H2-type zinc finger proteins revealed conserved C2H2 domains, which are demarcated by box. **B** Subcellular localization studies in tobaco leaves demonstrated that the 35S::*MdZAT1*-GFP fusion protein was localized to the nucleus. **C** Y1H assays indicated that MdZAT1 interacts with the promoter of *MdMYB114*. **D** EMSAs further confirmed the direct binding of MdZAT1-HIS to a TTGGGT motif within the *MdMYB114* promoter. The biotin-labeled probes were used for detection, while unlabeled (cold) and mutant probes served as competitors. **E** Luciferase reporter assays showed that MdZAT1 suppresses the transcriptional activity of the *MdMYB114* promoter. **F** ChIP-qPCR assays demonstrated the in vivo binding of MdZAT1 to the *MdMYB114* promoter. **G** Fruit development was observed at 0, 6, 12, and 18 days. **H** The anthocyanin content and *MdZAT1* expression levels during the fruit coloration. **I** Y1H assays indicated that MdZAT1 also interacts with the promoter of *MdCHI*. **J** EMSAs confirmed the direct binding of MdZAT1-HIS to two TTGGGT motifs within the *MdCHI* promoter. The biotin-labeled probes for detection and unlabeled (cold) and mutant probes as competitors. **K** Luciferase reporter assays demonstrated that MdZAT1 suppresses the transcriptional activity of the *MdCHI* promoter. **L** ChIP-qPCR assays confirmed the in vivo binding of MdZAT1 to the *MdCHI* promoter. **M** Y1H assays also indicated an interaction between MdZAT1 and the promoter of *MdANS*. **N** EMSAs confirmed the direct binding of MdZAT1-HIS to two TTGGGT motifs within the *MdANS* promoter. The biotin-labeled probes for detection and unlabeled (cold) and mutant probes as competitors. **O** Luciferase reporter assays showed that MdZAT1 suppresses the transcriptional activity of the *MdANS* promoter. **P** ChIP-qPCR assays confirmed the in vivo binding of MdZAT1 to the *MdANS* promoter. **Q** MdZAT1 directly represses anthocyanin biosynthesis by binding to the promoters of *MdCHI* and *MdANS,* and potentially regulates anthocyanin biosynthesis through *MdMYB114*. Data are presented as mean ± standard deviation (SD) from three biological replicates (*n* = 3). Asterisks indicate statistically significant differences based on Student’s t-tests (*p < 0.05, **p < 0.01)

Further, a Y1H assay was carried out to verify interaction between MdZAT1 and *MdMYB114* promoter. The yeast cells containing *MdZAT1*-pGADT7 and *MdMYB114*-pHIS2 grew on -Trp/-His/-Leu while those carrying the *MdMYB114*-pHIS2 and pGADT7 constructs did not (Fig. 1C). The result suggested that MdZAT1 interacts with the promoter of *MdMYB114*. Further, EMSA was performed to identify whether MdZAT1 can directly bind to the *MdMYB114* promoter. FEMU2, a C2H2 zinc finger protein, bound to the (G/T)TTGG(G/T)(G/T)T sequence in *Chlamydomonas reinhardtii* (Deng et al. 2014). We detected two TTGGGT binding sites in the *MdMYB114* promoter, and EMSAs indicated that MdZAT1 could bind directly to the *MdMYB114* promoter through probe 2 (Fig. 1D). To confirm that MdZAT1 could bind to the promoter of *MdMYB114* in vivo, we performed ChIP-qPCR assays. The M2 region was significantly enriched in MdZAT1-OE transgenic calli (Fig. 1E). The LUC reporter assay was performed to explore the transcriptional activity of MdZAT1 on *MdMYB114* promoter using the effector 35S:*MdZAT1* and the reporter *pMdMYB114*-LUC. When *pMdMYB114*-LUC was coexpressed with pGreen62-SK-*MdZAT1* in tobacco leaves, the luminescence signals were weaker than those of the control, indicating that MdZAT1 downregulated *MdMYB114* expression (Fig. 1F). These results suggested that MdZAT1 could suppress the expression of *MdMYB114* through directly binding to its promoter.

Further, to explore the relationship between MdZAT1 and anthocyanin biosynthesis, the content of anthocyanin and the expression of *MdZAT1* were measured at four stages of apple development (Fig. 1G). The anthocyanin content increased gradually with fruit development, while the expression of *MdZAT1* decreased (Fig. 1H), suggesting that MdZAT1 may be a negative regulator in anthocyanin pathway. The transcription levels of *MdMYB1*, *MdbHLH3*, *MdCHI*, *MdCHS*, *MdDFR*, and *MdANS* were the highest at 6 DABR, while those of *MdbHLH33*, *MdF3H*, and *MdUFGT, MdGST* were the highest at 12 DABR (Fig. S2A and B). The expression pattern of *MdMYB114* was consistent with anthocyanin content, but negatively correlated to *MdZAT1* expression (Fig. S2B).

We further transformed apple calli with the 35S::*MdZAT1*-GFP construct to analyze the function of MdZAT1 (Fig. S3A). PCR was performed to test the transgenic calli at the DNA and transcript levels to confirm positive lines (Fig. S3B and C). A decrease in anthocyanin content in the *MdZAT1* overexpressing lines was observed compared with WT (Fig. S3D). Meanwhile, the expression levels *MdCHS*, *MdCHI*, *MdANS*, *MdbHLH3*, *MdMYB1* and *MdMYB114*, were significantly downregulated in the *MdZAT1*-OE calli (Fig. S3E).

To investigate the function of MdZAT1 in apple peels, we suppressed MdZAT1 expression using VIGS system to verify the function of MdZAT1. The apple peels injected with pTRV1 and empty pTRV2 showed a normal phenotype, while those with pTRV1 and *MdZAT1*-pTRV2 had an increase in anthocyanin content (Fig. S4A and B). In the apple peels with pTRV1 and *MdZAT1*-pTRV2, the expression levels of the anthocyanin biosynthesis-associated related genes were upregulated (Fig. S4C). The peel of apple fruit with overexpression of *MdZAT1* showed a pale appearance, while the control showed a normal phenotype (Fig. S5A). The content of anthocyanin was lower in apple peel injected with *MdZAT1*-pRI101 compared to pRI101 (Fig. S5B). The transcript abundances of anthocyanin related genes were also inhibited with *MdZAT1*-pRI101 in apple peel (Fig. S5C). These results confirmed that MdZAT1 was a negative regulator in the accumulation of anthocyanin.

Finally, according to the expression of related genes in transgenic materials, we tested whether MdZAT1 binds to the genes *MdCHS*, *MdCHI*, *MdF3H*, *MdDFR*, *MdANS*, *MdUFGT*, *MdMYB1*, MdGST, *MdbHLH3* and *MdbHLH33* promoters. Y1H assays were performed to investigate the potential interaction of MdZAT1 with those promoters. The Y1H results showed that MdZAT1 could interact with *MdCHI* and *MdANS* promoters but not with other promoters (Fig. 1I, M and S6).

Further, EMSA was performed t and the result showed that MdZAT1 bound to the *MdCHI* promoter. Subsequently, LUC reporter assay was performed using the 35S:MdZAT1 effector and the *pMdCHI*-LUC reporter vectors. Relative to the control, LUC luminescence signals were weaker and the *MdCHI* activity was inhibited when *pMdCHI*-LUC was coexpressed with pGreen62-SK-*MdZAT1* in tobacco leaves, indicating that MdZAT1 downregulated *MdCHI* expression (Fig. 1K). To confirm that MdZAT1 could bind to the promoter of *MdCHI* in vivo, we performed ChIP-qPCR assays. The C1 region was significantly enriched in *MdZAT1*-OE transgenic calli, indicating that MdZAT1 directly bind to *MdCHI* promoter in vivo (Fig. 1L). We identified two fragments in the *MdANS* promoter containing the TTGGGT motif and EMSA results showed that MdZAT1 bound to probe 1 and probe 2 (Fig. 1N), indicating that MdZAT1 could bind to the *MdANS* promoter. Subsequently, LUC reporter assay was performed using the 35S:MdZAT1 effector and the *pMdANS*-LUC reporter vectors. Relative to the control, LUC luminescence signals were weaker and the *MdANS* activity was inhibited when *pMdANS*-LUC was coexpressed with pGreen62-SK-*MdZAT1* in tobacco leaves, indicating that MdZAT1 downregulated *MdANS* expression (Fig. 1O). ChIP-qPCR assays indicated that MdZAT1 directly bind to *MdANS* promoter in vivo (Fig. 1P). Taken together, MdZAT1 could directly bind to the promoters of *MdCHI* and *MdANS* and repressed their expression to decrease anthocyanin accumulation.

In summary, our results demonstrated that MdZAT1, a C2H2-type zinc finger protein, could repress anthocyanin biosynthesis through inhibiting the transcriptional activity of *MdCHI, MdANS* and *MdMYB114* by binding to the TTGGGT motif (Fig. 1Q). Our research provides insights into the molecular mechanism via C2H2-type zinc finger protein in regulating anthocyanin accumulation.

## Supplementary Information

Supplementary Material 1.

Supplementary Material 2.

## Data Availability

The data and materials would be available upon reasonable request.
