# Supplementary material for: C2H2-type zinc finger protein transcription factor MdZAT1 plays a negative role in anthocyanin biosynthesis in apple
Source: Mol Hortic. 2025 May 8;5:28. doi: 10.1186/s43897-025-00150-6 (PMC12060325; doi:10.1186/s43897-025-00150-6)
Supplement: Supplementary file 1 — Supplementary Material 1. [file 43897_2025_150_MOESM1_ESM.docx]

**Figures and figure legends**


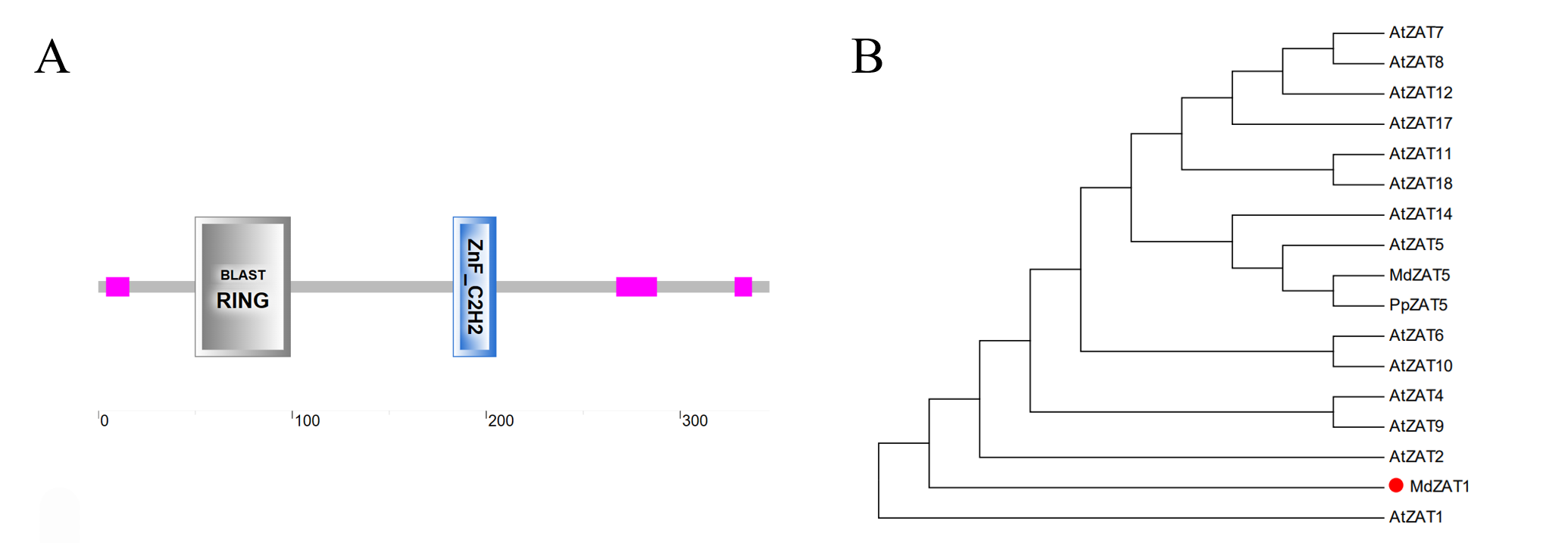


**Fig. S1. Domain analysis and phylogenetic analysis of MdZAT1** (A) MdZAT1 contains a C2H2-type zinc finger domain between amino acids 183 and 205. (B) Phylogenetic tree of the C2H2-type zinc finger protein sequences from different species. AtZAT1 (AT2G46800), AtZAT2 (AT2G17180), AtZAT5 (AT2G28200), AtZAT6 (AT5G04340), AtZAT7 (AT3G46090), AtZAT8 (AT3G46080), AtZAT9 (AT3G60580), AtZAT10 (AT1G27730), AtZAT11 (AT2G37430), AtZAT12 (AT5G59820), AtZAT14 (AT5G03510), AtZAT17 (AT2G28710), MdZAT5 (MD03G1128800), PpZAT5 (EVM0021417.1).


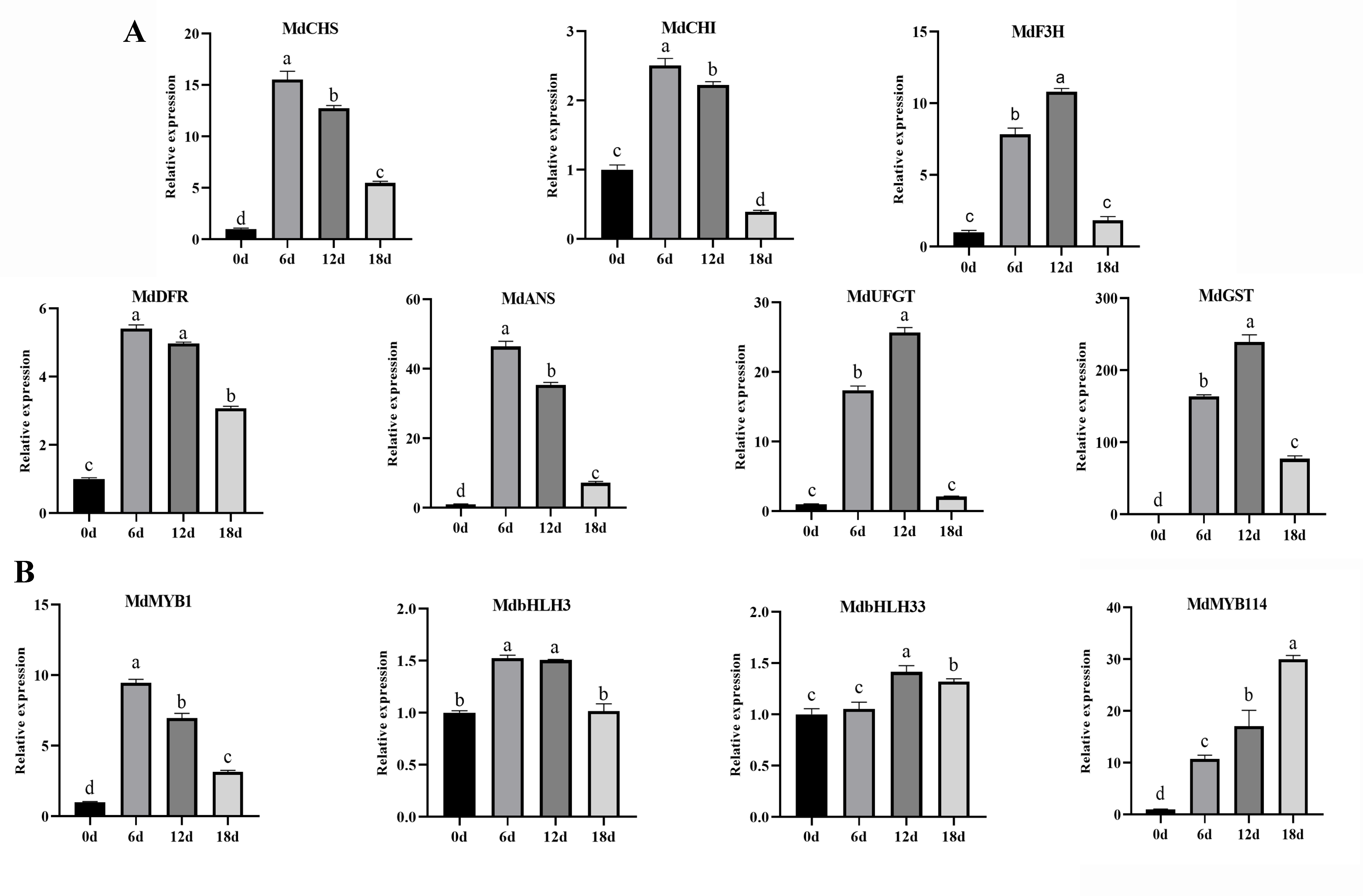


**Fig. S2. The related gene expression during the apple fruit development. The expression of** structural genes(A) and transcription factors(B) during anthocyanin accumulation in apple. Different letters representative significant differences between stages (Student’s *t*-test). The data shown are mean values ± SD (n = 3).


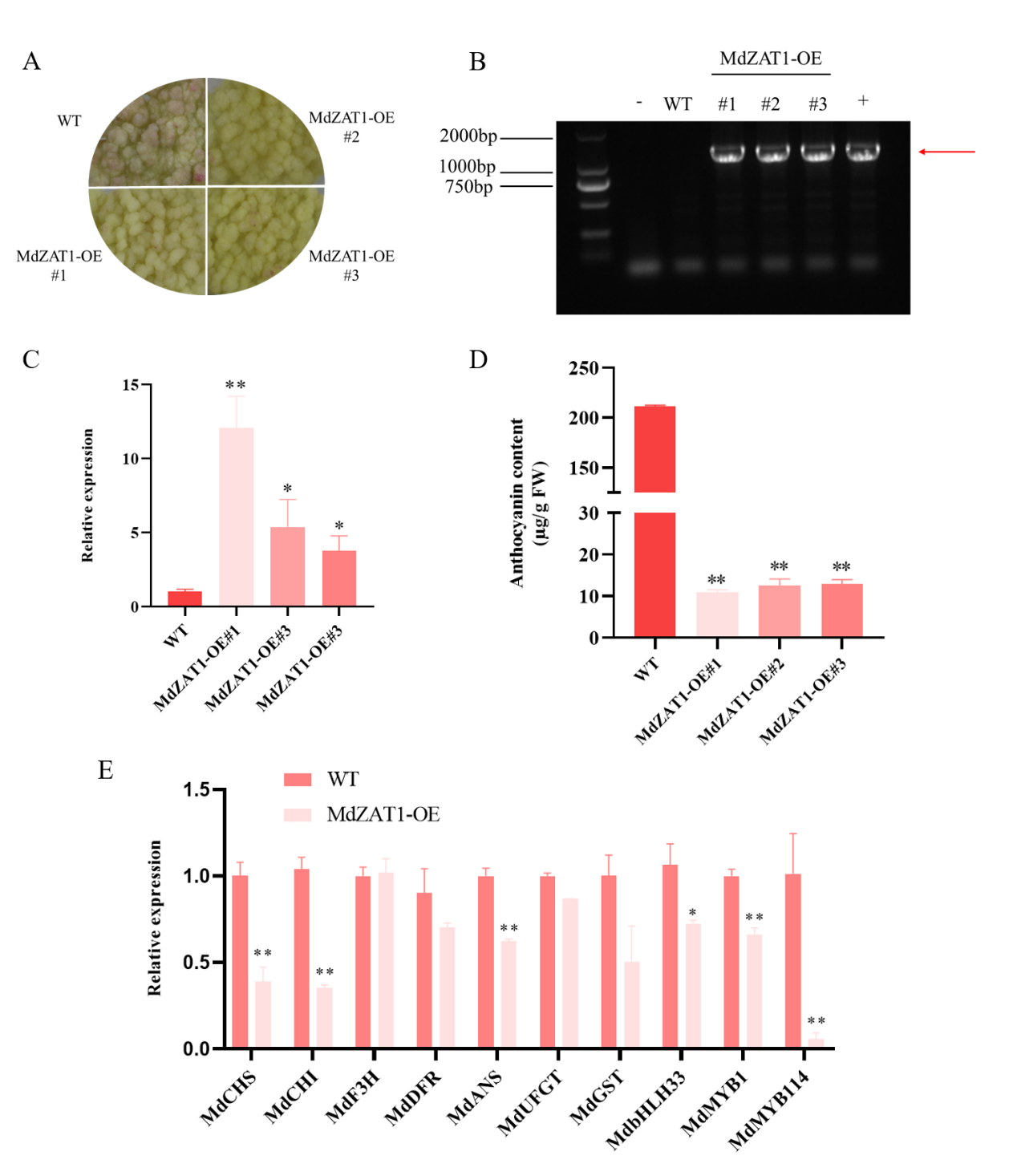


**Fig. S3. Functional characterization of *MdZAT1* in apple calli.** (A) Phenotypes of the 35S::GFP and the *MdZAT1*-OE apple calli. (B) PCR confirms the presence of transgene *MdZAT1*-OE calli at DNA level. The arrow refers to the amplicon strip. “-” represents a negative control (H_2_O), and “+” represents a positive control (*MdZAT1*-pRI101 plasmid). (C) qRT-PCR confirms the presence of transgene in the *MdZAT1*-OE calli at the transcription level. (D) Total anthocyanin content in the four apple calli. (E) Relative expression levels of genes of anthocyanin pathway in *MdZAT1*-OE calli and GFP calli. The data shown are mean values ± SD (n = 3). * representatives statistically significant differences (Student’s *t*-test, * representatives *p* <0.05; ** representatives *p* <0.01).


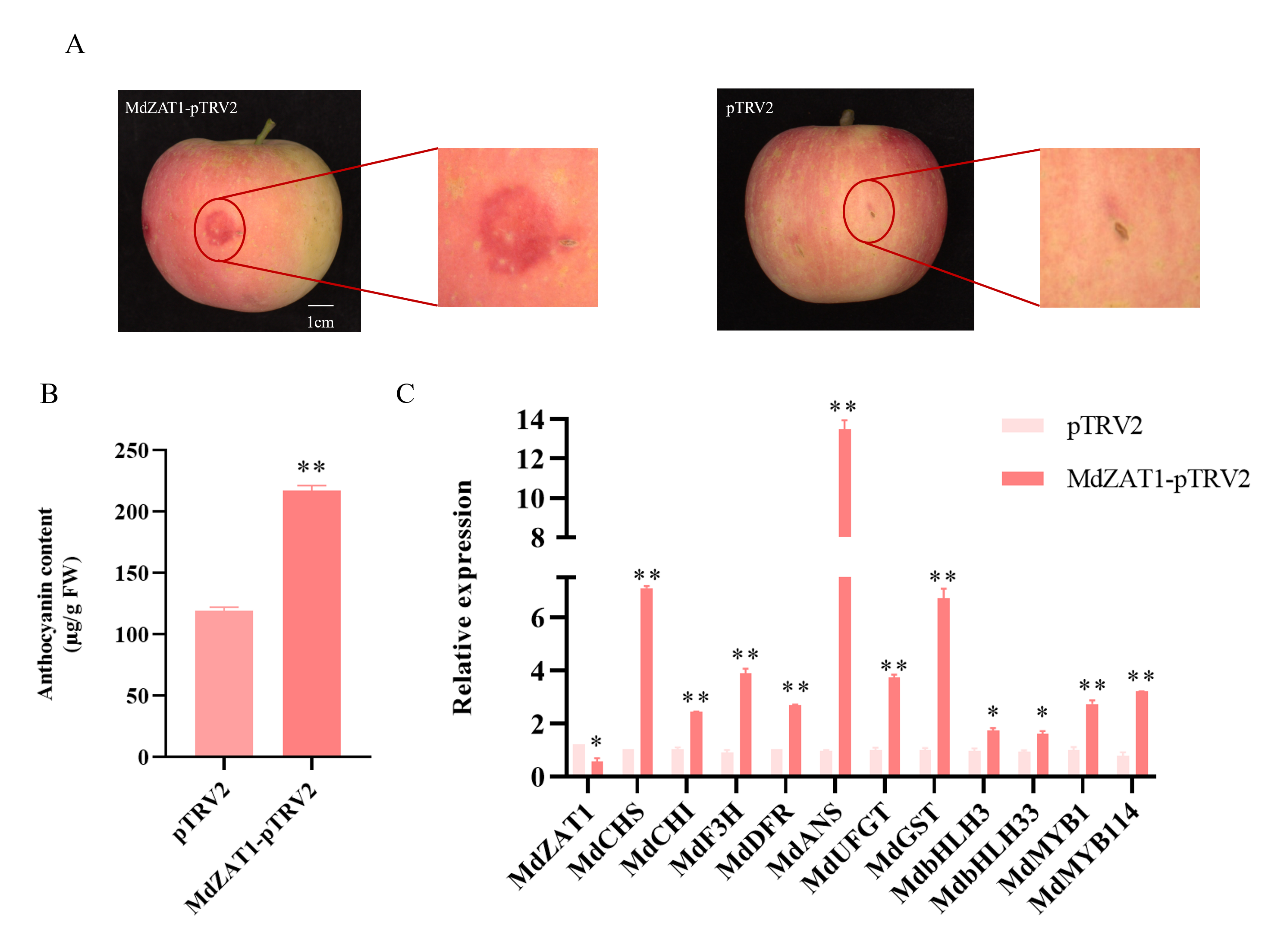


**Fig. S4. *MdZAT1* silencing promotes anthocyanin biosynthesis in apple peel.** (A) Phenotypes of apple fruits after *MdZAT1* silencing. The Empty pTRV2 with pTRV1 was the control. Bar = 1cm. (B) Anthocyanin content in the peels of *MdZAT1*-silenced and control apple fruits. (C) The expressions of anthocyanin-associated genes in *MdZAT1*-silenced and control apple. The data shown are mean values ± SD (n = 3). * representatives statistically significant differences (Student’s *t*-test, * representatives *p*<0.05; ** representatives *p* <0.01).


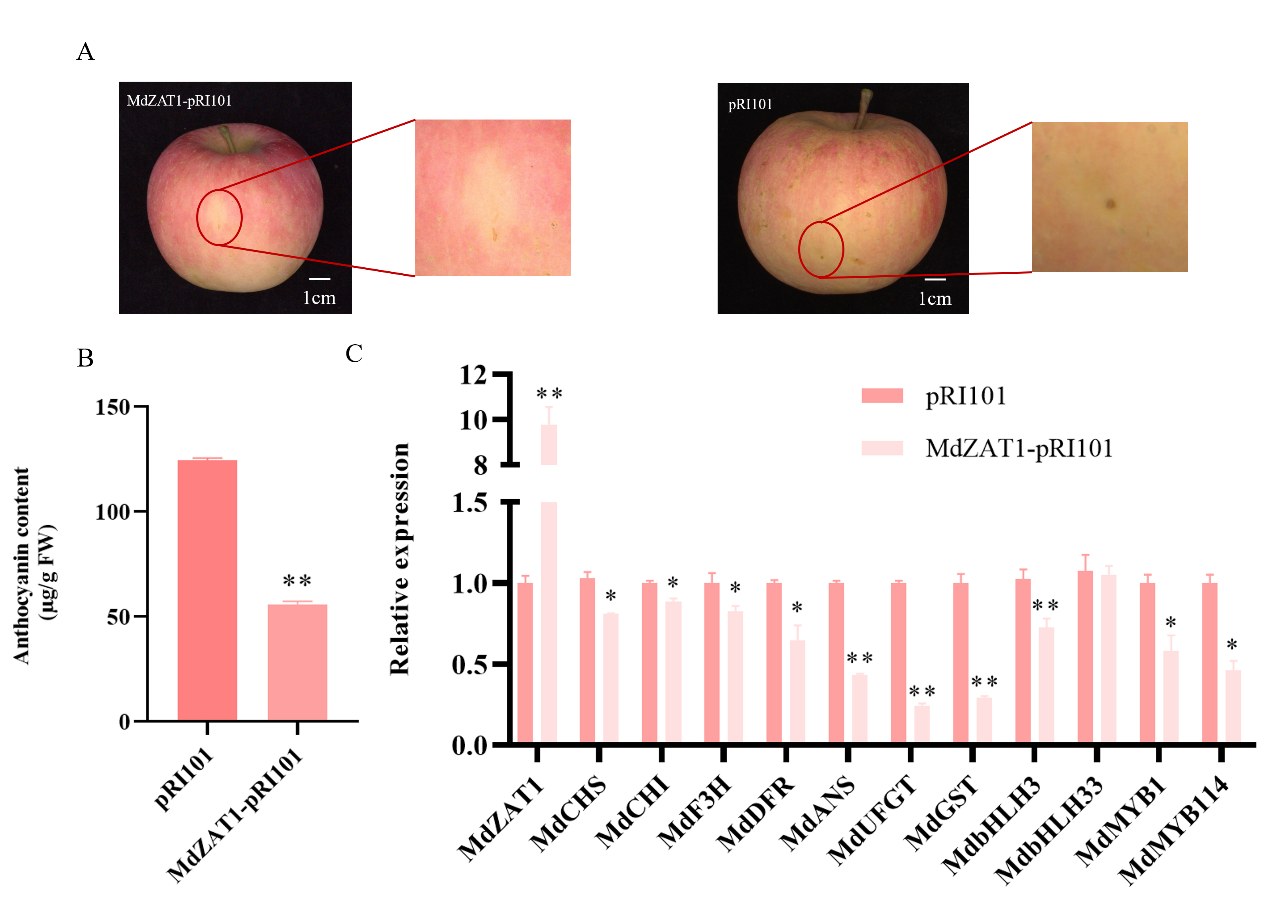


**Fig. S5. *MdZAT1* overexpression inhibits anthocyanin biosynthesis in apple peel.** (A) Phenotypes of apple fruits overexpressing *MdZAT1*. Empty pRI101 was the control. Bar = 1cm. (B) Anthocyanin content in the peels of *MdZAT1*-overexpressing and control apple fruits. (C) The expressions of anthocyanin biosynthesis-associated genes in *MdZAT1*-overexpressing and control apple. The data shown are mean values ± SD (n = 3). * representatives statistically significant differences (Student’s *t*-test, representatives, *p* <0.05; ** representatives *p* <0.01).


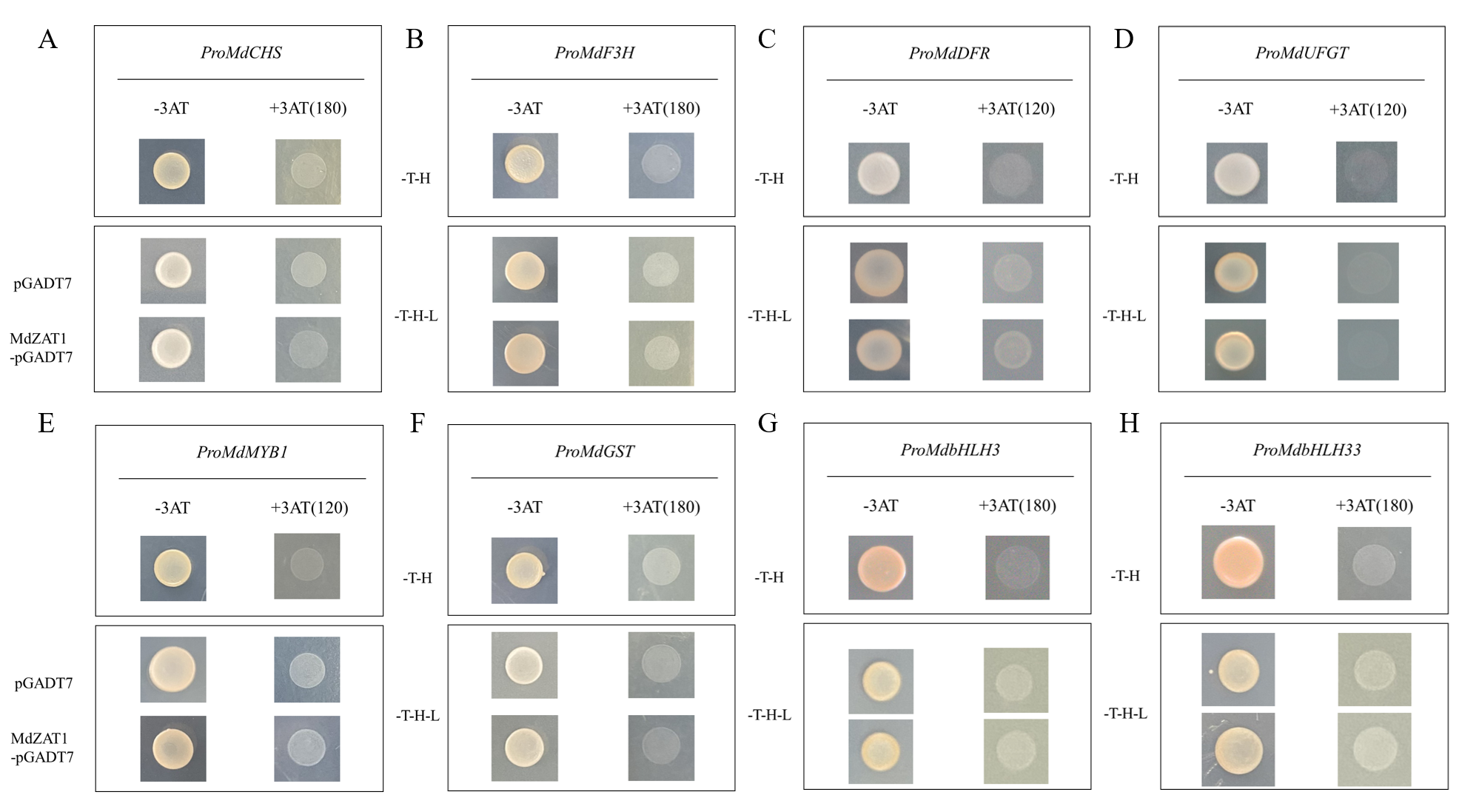


**Fig. S6.** Y1H assays indicate the interaction between MdZAT1 protein and the promoters of *MdCHS*, *MdF3H*, *MdDFR*, *MdUFGT*, *MdMYB1*, *MdGST*, *MdbHLH3 and MdbHLH33*.

**1. Materials and methods**

**1.1 Plant material**

‘Fuli’ apple was used as experimental material and it was planted in Qingdao of Shandong Province. On September 28, 2022, we removed the bags of fruits and fruits were sampled at four development stages, they are S1-S4 (0 day after bag removal (DABR), 6 DABR, 12 DABR, 18 DABR). We collected more than ten fruits at S1-S4 respectively, and then pared the peels into the liquid nitrogen and ultimately stored in ultra-low temperature refrigerator until use.

‘Orin’ calli was used as experimental material and cultured on Murashige and Skoog (MS) medium in the dark at 25 ℃. The MS included 0.4 mg/L 6-benzylamino-purine and 1.5 mg/L 2,4-dichlorophenoxyacetic acid.

**1.2 Measurement of anthocyanin content**

We used the HCl-methanol method to extract the anthocyanin from the samples. The sample (0.5 g) was ground into powder with liquid nitrogen, and anthocyanin was extracted from the obtained powder with 15 mL hydrochloric acid-methanol (10%) for 1day at 4 °C in the dark. After centrifugation, the supernatant (1 mL) was respectively mixed with 4 mL of KCL buffer (0.025M; pH1.0), 4 mL of NaAc buffer (0.4M; pH4.5), respectively. The solution was incubated at 4 °C for 0.25h. Finally, the absorbance of the solution was measured by a spectrophotometer at 510 nm and 700 nm, and the calculation formula of the anthocyanin content is:

ΔA*5*0.005*1000*449.2/ (29600*0.5)

Where, ΔA=(A510-A700) *1.0-(A510-A700) *4.5

**1.3 Phylogenetic analysis**

We performed phylogenetic analysis by using the MEGA 5.1 software and set the parameter was 1000 of bootstrap value. Aligned amino acid sequences of the proteins using DNAMAN software. All the protein sequences were obtained from NCBI (www.ncbi.nlm.nih.gov) and all the IDs of these proteins were listed in Table S1.

**1.4 Total RNA isolation and quantitative real-time PCR (RT-PCR)**

We extracted the total RNA from the samples including apple peels and calli using the RNA prep Pure Plant Plus Kit (DP441, TIANGEN, Beijing, China). The cDNAs were synthesis by using the Prime Script first-strand cDNA Synthesis Kit (Takara). Then, we used the cDNAs as templates to perform RT-PCR on the Fluorescence quantitative PCR instrument (QuantStudio5). The primers used in this assay are showed in this paper (Table S2). Three independent biological and technical replicates respective were maintained in the assay. The relative expression levels of genes were calculated used the 2^-ΔΔCT^ method(Livak and Schmittgen 2001).

**1.5 Genetic transformation**

We cloned the coding sequence (CDS) of *MdZAT1* into the pRI101 vector to obtain *MdZAT1-*pRI101 recombinant vector and then transformed it into GV3101 *Agrobacterium tumefaciens* strain (WEIDI, Shanghai, China) following the steps in the manual. Further, ‘Orin’ apple calli were incubated with the cells of *Agrobacterium tumefaciens* which carrying the *MdZAT1-*pRI101vector for 30 minutes, spread on MS culture medium. After co-culturing at 24 °C in the dark for two days, transfer the transgenic calli lines to MS medium containing 50mg/L Kanamycin for cultivation.

**1.6 Virus-induced gene silencing**

The 400 bp (451-850bp) region of the *MdZAT1* CDS was cloned into the pTRV2 vector to construct *MdZAT1*-pTRV2 vector. Three vectors including MdZAT1-pTRV2, pTRV1, and pTRV2, were transformed into GV3101 *Agrobacterium tumefaciens* strain (WEIDI, Shanghai, China) for the VIGS experiments, respectively. After culturing, the *Agrobacterium tumefaciens* cells harboring pTRV2-*MdZAT1*, pTRV2, and pTRV1 were resuspended in the buffer to an OD_600_ of 0.8. The buffer contained 10 mM MgCl_2_, 10 mM MES, and 150 μM acetosyringone (AS). Then the suspension was stored at 24 °C for 2 hours. The *Agrobacterium tumefaciens* pTRV1 and pTRV2-*MdZAT1* were mixed using the ratio 1:1 and injected into the apple peel following the previous method(Jiang et al. 2019), using the 1:1 mixture of *Agrobacterium tumefaciens* pTRV1 and pTRV2 as the control. Observed the phenotype of the fruit and collected 3 to 5 days after injection.

**1.7 Transient overexpression of MdZAT1 in apple fruits**

The *Agrobacterium tumefaciens* cells carrying the *MdZAT1*-pRI101 construct were used to transform the apple peel. The phenotype of the fruit was analyzed and collected 3 to 5 days after transformation.

**1.8 Subcellular localization**

We cloned the CDS of *MdZAT1* was cloned into the pCAMBIA2300 vector, and the obtained pCAMBIA2300-*MdZAT1*-EGFP vector was transformed into GV3101 *Agrobacterium tumefaciens* strain (WEIDI, Shanghai, China). Then the *Agrobacterium tumefaciens* cells harboring pCAMBIA2300-*MdZAT1*-EGFP were injected into *Nicotiana benthamiana* leaves; the cells carrying pCAMBIA2300-EGFP were injected to the leaves and maintained as the control. Two to three days after transformation, the fluorescence signals were analyzed using the imaging system (EVOS FL AUTO 2.0).

**1.9 Luciferase reporter assay (LUC)**

We cloned the CDS of *MdZAT1* into the pGreenII 62-SK vector and cloned the promoters of *MdMYB114* and *MdANS* into the pGreenII 0800-LUC vector. All these vectors were transformed into GV3101 *Agrobacterium tumefaciens* strain (WEIDI, Shanghai, China). According to the method of Cong et al., the *Agrobacterium tumefaciens* cells harboring the pGreenII 62-SK vector and pGreenII 0800-LUC vector were injected into *N. benthamiana* leaves. After 2 days, the fluorescence signals were detected using a Living Plant fluorescence detector (Newton7.0). The ratio of firefly luciferase to Renilla luciferase was determined using the Dual Luciferase Reporter Assay Kit (Vazyme, Nanjing, China).

**1.10 Y1H assay**

The promoters were respectively inserted into the pHIS2 vector, while the CDS of *MdZAT1* was cloned into the pGADT7 vector. First, the promoters*-*pHIS2 vectors were respectively transformed into the yeast Y187 strain and allowed to grow on -Trp/-His (-T/-H) medium with different concentrations of 3-amino-1, 2, 4-triazole (3-AT) to determine the 3-AT optimal concentration to suppress background histidine leakiness of pHIS2. Then, cells carrying both vectors were transferred into the corresponding culture medium to observe the growth of the plaque. In this section, the empty pGADT7 vector was used as the negative control.

**1.11 EMSA**

We cloned the CDS of *MdZAT1* into the pET32a vector, and then transformed the *MdZAT1*-pET32a vector Rosetta-gami (DE3) to induce the expression of the fusion protein. The Ni-NTA 6FF Sefinose (TM) Resin Kit (Sangon Biotech, Shanghai, China) was used to purify the fusion protein. The probes were labeled by biotin at the 3’ end for EMSA and obtain from Sangon Biotech Co. Ltd. (Shanghai, China) and annealed to the complementary oligonucleotides using Annealing Buffer for DNA Oligos (5×) (Beyotime Biotechnology, Shanghai, China). Finally, the purified protein was incubated with the probe in darkness at 24 ℃ for 30 minutes for EMSA. The luminescence was determined used the BeyoECL Plus Kit according to the instruction (Beyotime Biotechnology, Shanghai, China).

**1.12 ChIP-qPCR**

We used the anti-GFP antibody and the ChIP Assay Kit (Beyotime Biotechnology, Shanghai, China) for the ChIP assays according the previous study(Jiang et al. 2020). The results were analyzed by qPCR. All primers used are listed in Table S2.

**References**

Livak KJ, Schmittgen TD (2001) Analysis of relative gene expression data using real-time quantitative PCR and the 2(-Delta Delta C(T)) Method. Methods 25 (4):402-408.

Jiang S, Wang N, Chen M, Zhang R, Sun Q, Xu H, Zhang Z, Wang Y, Sui X, Wang S, Fang H, Zuo W, Su M, Zhang J, Fei Z, Chen X (2020) Methylation of MdMYB1 locus mediated by RdDM pathway regulates anthocyanin biosynthesis in apple. Plant Biotechnol J 18 (8):1736-1748.
