# Supplementary material for: C2H2-type zinc finger protein transcription factor MdZAT1 plays a negative role in anthocyanin biosynthesis in apple
Source: Mol Hortic. 2025 May 8;5:28. doi: 10.1186/s43897-025-00150-6 (PMC12060325; doi:10.1186/s43897-025-00150-6)
Supplement: Supplementary file 2 — Supplementary Material 2. [file 43897_2025_150_MOESM2_ESM.docx]

**Table S1. Sequences used for phylogenetic trees and sequence alignment.**

>MdZAT1 (XP_028950153.1)

MPKDRRGRSVTPDRYRASPYPCSSSHTRRSSPRIPSETEDNVKEWEEARCPVCLEHPHNAVLLICSSYEQGCRPYMCDTSYRHSNCLDLYCKSFSAETSPTIPPEGAAQISDTQSSPSATLESTITQVQNDSTIEEVLSSMNAVSCEHQADPKLVCPLCRGEITDWIIVESARCFMNAKSRNCSCETCNYSGTYADLRKHARLEHPLVCPSEADPERQRTWRSLERQRDIGDLFSSIQSSVGEDRGDDSSSLPADDGAGWLTIFFLVRVVRPGSSSRSSSWSGTTRTRAQVSMRRRATRLWGESYEGEAASSTLEEDNESSDGDSGVRRRRSARLRRWTTPDNNQP

>MbZAT1 (TQD97147.1)

MPKDRRGRSVTPDRYRASPYPCSSSHTRRSSPRIPSETEDNVKEWEEARCPVCLEHPHNAVLLICSSYEQGCRPYMCDTSYRHSNCLDQYCKSFSAETAPTIPPEGAAQISDTQSSPSATLESTITQVQNDRTIEEVLSSMNAVSCEHQADPKLVCPLCRGEITDWIIVESARCFMNAKSRNCSCETCNYSGTYADLRKHARLEHPLVCPSEADPERQRTWRSLERQRDIGDLFSSIQSSVGEDRGDDSSSLPADDGAGWLTIFFLVRVVRPGSSSRSSSWSGTTRTRAQVSMRRRATRLWGESYEGEAASSTLEEDNESSDGDSGVRRRRSARLRRWTTPDNNQP

>PcZAT1 (KAB2618672.1)

MPKERRGRSVTPDRYRASPYPCSSSHTRWSSPRIPSETEDNVKEWEEARCPVCLEHPHNAVLLICSSYEQGCRPYMCDTSYRHSNCLDQYCKSFSAETSPTIPPEGATQISDTQSSPSATLESTITQVQNDRTIEEVLSSMNAVSCEHQADPKLVCPLCRGEITDWIIVESARCFMNAKSRNCSCETCNYSGTYADLRTHARLEHPLVCPSEADPERQRTWRSLERQRDIGDLFSSIQSSVGEDRGDDSSSLPADDGAGWLTIFFLVRVVRPGSSSRSSSWSGTTRTRAQVSMRRRATRLWGESYEGEAASSTLEEDNESSDGDSGVRRRRSARLRRWTTPDNNQP

>AtSIZ1 (AT3G25910.1)

MPKERKERSVSLDKYKRSPLCCEASLALKPSEKQVKEWEEARCPVCMEHPHNGILLICSSYENGCRPYMCDTSHRHSNCFDQFRKASKEKPSLSLLREEEESNEPTEMEDVDSDSTAVNLLGEAASEITVVDLSDGERGEEEVEEEEEEVVVEEEEEGIVTTEEDQEKNKPQKLTCPLCRGHIKEWVVVKAARCFMNSKHRSCSCETCDFSGSYSDLRKHARLLHPGVRPSEADPERQRSWRRLERQSDLGDLLSTLQSSFGGDEISNDDGFLFADTRLLTVYFLIRVFRPESSGSRSSSWSGTSRARTHTSGRRRSSRPASLWGESYEGNTGTSPRDEENNQSSDEQVSGTRRRRSRRRTVIDDDDEEEEP

>PpZAT1 (XP_020424729.1)

MPKDRRGRSVSRDRYRASPYPCSSSHTRRPPPKIPSETEENVKEWEDTRCPVCLEHPHNAVLLICSSYEKGCRPYMCDTSYRHSNCLDQYCKSFSAETSPTVPPEGENQILDTQSSPAETLESTITHVQNDRIEEEAPTMNPIACEHEAQPKLLCPLCRGEIKDWIIVEPARCFMNAKSRNCSCETCSFIGTYADLRNHARLEHPQARPSEADPERQRTWRSLERQRDFGDLLSTLQSSIGEDRGDDSSLPVDDGGWLTVVFLVRVIQPGASLRSSTWSGPRRTRAQVRMRGRATTLWGESYDGEAASASREEDNDSSDGSTGARRRSARLRRRTTPDNQP

>FvZAT1 (XP_004287360.1)

MPKERRDRSVSRERYRASPYPCSSSHTKRPSLKIPSETEENVKEWEEARCPVCLEHPHNAVLLICSSFERGCRPYMCDTSYRHSNCLDQFCKSFSEEDPPEILPQEETLLSDNLLPPFETSESIITDVQTDITEEEPPPMIPISCEHQEQPKLVCPLCRGEVKDWIIVEPARCFMNAKLRNCSCETCSFSGTYTDLRKHARLEHPQTRPSEADPERQRAWRSLERQRDIGDLLSTLQSSTGEDRGDDSSLAVEHDGGGWLTVFFLVRIYRPTSGSSSRSSSWSPTTRTRAQISMRRRATRLWGESYESEAASSSREADNDSSDGGSAPTRRQSARLRQRRRTTPDNNQP

>VvZAT1 (XP_002285038.1)

MPKDRRDRSVSFDRYRASPFPCSSSRGKRSSPKKSSETEKEVKEWEDARCPVCMEHPHNAVLLLCSSNDKGCRPYMCDTSYRHSNCLDQFRKSFSESSSTVPLQEEMPPSDTQLSPMVTSEATDVDLHGERSEEGPFTMHTMSCENKTQPKLVCPLCRGQINGWTVVEPARHFMNAKSRSCACETCDFSGTYTDLRKHARLEHPLVRPSEADPERQRNWRRMERQRDLGDLLSTLQSSFGEERGDDSILPIDEGGWLTVFFLIRVFRPSSSSRSSSWSGTSRARAQLTIRRRSTRLWGESYEAESGSASRDEDNESSDGGSGPWRHARPQRRTTPDNEP

>MdZAT5(MD03G1128800)

MEGQEELVVTNDQASQMIMIKGKRTKRQRPQSPNGLVTAAVTSSSSSACGAGTIGGGDHDYNYYGNSFTSPTTSGEIYESTEEEEDMANCLILLAQGYHVNPKQTIEERLAQNTNMGKAGFFVYECKTCNRTFPSFQALGGHRASHKKPKSMSSTEEMIKKSPPAAAPPTHHFITATTFEEFEDQSKQLIKYKSSPPPAIPIQVGNKPKIHECSICRSEFTSGQALGGHMRRHRTASAATNSNTISGSATATHVAVNNSSNNMIGSSTKLQRNVLPLDLNLPAPEDHDHHHHHHHRESKFQFVPTQQTTLVFNAPALVDCHY

>AtZAT6(AT5G04340)

MALETLTSPRLSSPMPTLFQDSALGFHGSKGKRSKRSRSEFDRQSLTEDEYIALCLMLLARDGDRNRDLDLPSSSSSPPLLPPLPTPIYKCSVCDKAFSSYQALGGHKASHRKSFSLTQSAGGDELSTSSAITTSGISGGGGGSVKSHVCSICHKSFATGQALGGHKRCHYEGKNGGGVSSSVSNSEDVGSTSHVSSGHRGFDLNIPPIPEFSMVNGDEEVMSPMPAKKLRFDFPEKP

>PpZAT5(EVM0021417.1)

MEGQEELVVTNEQASQMSMIKGKRTKRQRPQSPNGVVTAAVTSSSSSACGVATIGGGDHEYNYYGSSFTSPTTSSEIYESTEEEEDMANCLILLAQGDHGNPKQIIEERLAQNINMGKAGFLVYECKTCNRTFPSFQALGGHRASHKKPKSMSSTEEIIKKLPPPAAAPPTLHSITATTFEKFEDQSKQFIKYKSSPSPAIPIQIGNKPKIHECSICGSEFTSGQALGGHMRRHRTASAATNSNSTSGGPGGATATHVAVSNSSNDMIGTSTKLQRNVLPLDLNLPAPEDHDHHHHHHRESKFQFVPTQQTTLVFNAPALVDCQY

>AtZAT11(AT2G37430)

MKRERSDFEESLKNIDIAKCLMILAQTSMVKQIGLNQHTESHTSNQFECKTCNKRFSSFQALGGHRASHKKPKLTVEQKDVKHLSNDYKGNHFHKCSICSQSFGTGQALGGHMRRHRSSMTVEPSFISPMIPSMPVLKRCGSSKRILSLDLNLTPLENDLEYIFGKTFVPKIDMKFVL

>AtZAT7(AT3G46090)

MVARSEEIVIVEEDTTAKCLMLLSRVGECGGGCGGDERVFRCKTCLKEFSSFQALGGHRASHKKLINSDNPSLLGSLSNKKTKTSHPCPICGVKFPMGQALGGHMRRHRNEKVSGSLVTRSFLPETTTVTALKKFSSGKRVACLDLDLDSMESLVNWKLELGRTISWS

>AtZAT10(AT1G27730)

MALEALTSPRLASPIPPLFEDSSVFHGVEHWTKGKRSKRSRSDFHHQNLTEEEYLAFCLMLLARDNRQPPPPPAVEKLSYKCSVCDKTFSSYQALGGHKASHRKNLSQTLSGGGDDHSTSSATTTSAVTTGSGKSHVCTICNKSFPSGQALGGHKRCHYEGNNNINTSSVSNSEGAGSTSHVSSSHRGFDLNIPPIPEFSMVNGDDEVMSPMPAKKPRFDFPVKLQL

>AtZAT1(AT2G46800)

MESSSPHHSHIVEVNVGKSDEERIIVASKVCGEAPCGFSDSKNASGDAHERSASMRKLCIAVVLCLVFMSVEVVGGIKANSLAILTDAAHLLSDVAAFAISLFSLWAAGWEATPRQTYGFFRIEILGALVSIQLIWLLTGILVYEAIIRIVTETSEVNGFLMFLVAAFGLVVNIIMAVLLGHDHGHSHGHGHGHGHDHHNHSHGVTVTTHHHHHDHEHGHSHGHGEDKHHAHGDVTEQLLDKSKTQVAAKEKRKRNINLQGAYLHVLGDSIQSVGVMIGGAIIWYNPEWKIVDLICTLAFSVIVLGTTINMIRNILEVLMESTPREIDATKLEKGLLEMEEVVAVHELHIWAITVGKVLLACHVNIRPEADADMVLNKVIDYIRREYNISHVTIQIERVLLACHVNIRPEADADMVLNKVIDYIRREYNISHVTIQIERKLSKIRVFVSAFSLTIKSIKFLHFSL

>AtZAT17(AT2G28710)

MERGRSDMEMINNMANCLILLSKAHQNDTKSRVFACKTCNKEFPSFQALGGHRASHRRSAALEGHAPPSPKRVKPVKHECPICGAEFAVGQALGGHMRKHRGGSGGGGGRSLAPATAPVTMKKSGGGNGKRVLCLDLNLTPLENEDLKLELGRFIF

>AtZAT12(AT5G59820)

MVAISEIKSTVDVTAANCLMLLSRVGQENVDGGDQKRVFTCKTCLKQFHSFQALGGHRASHKKPNNDALSSGLMKKVKTSSHPCPICGVEFPMGQALGGHMRRHRNESGAAGGALVTRALLPEPTVTTLKKSSSGKRVACLDLSLGMVDNLNLKLELGRTVY

>AtZAT14(AT5G03510)

MTSVLEESGDHARLVLLIKGKRTKRQRSASPLMMNAAAVSSVCSGERSSVEVREEEAAGEVEFQGATDEDEDMANCLILLSQGHQAKSSDDHLSMQRMGFFSNKKPVASLGLGLDGVYQCKTCDKSFHSFQALGGHRASHKKPKLGASVFKCVEKKTASASTVETVEAGAVGSFLSLQVTSSDGSKKPEKTHECSICKAEFSSGQALGGHMRRHRGLTINANATSAIKTAISSSSHHHHEESIRPKNFLQLDLNLPAPEDERCCEKPKFAFASKDQILLFTAASNSLIDCHH

>AtZAT2(AT2G17180)

MSNTSNSDPNSDIPFASSNVTLPSYNQNPRRKRTKLTNNEVGSSSSSPRPKPVTQPDPDASQIARPCTECGKQFGSLKALFGHMRCHPERQWRGINPPSNFKRRINSNAASSSSSWDPSEEEHNIASCLLMMANGDVPTRSSEVEERFECDGCKKVFGSHQALGGHRATHKDVKGCFANKNITEDPPPPPPQEIVDQDKGKSVKLVSGMNHRCNICSRVFSSGQALGGHMRCHWEKDQEENQVRGIDLNVPAATSSDTTLGCSLDLRLGL

>AtZAT5(AT2G28200)

MMMGQDEVGSDQTQIIKGKRTKRQRSSSTFVVTAATTVTSTSSSAGGSGGERAVSDEYNSAVSSPVTTDCTQEEEDMAICLIMLARGTVLPSPDLKNSRKIHQKISSENSSFYVYECKTCNRTFSSFQALGGHRASHKKPRTSTEEKTRLPLTQPKSSASEEGQNSHFKVSGSALASQASNIINKANKVHECSICGSEFTSGQALGGHMRRHRTAVTTISPVAATAEVSRNSTEEEIEINIGRSMEQQRKYLPLDLNLPAPEDDLRESKFQGIVFSATPALIDCHY

>AtZAT8(AT3G46080)

MVARSEEVEIVEDTAAKCLMLLSRVGECGGGGEKRVFRCKTCLKEFSSFQALGGHRASHKKLINSSDPSLLGSLSNKKTKTATSHPCPICGVEFPMGQALGGHMRRHRSEKASPGTLVTRSFLPETTTVTTLKKSSSGKRVACLDLDSMESLVNWKLELGRTIS

>AtZAT9(AT3G60580)

MESYKCRVCFKSFVNGKALGGHMRSHMSNSHEEEQRPSQLSYETESDVSSSDPKFAFTSSVLLEDGESESESSRNVINLTRKRSKRTRKLDSFVTKKVKTSQLGYKPESDQEPPHSSASDTTTEEDLAFCLMMLSRDKWKKNKSNKEVVEEIETEEESEGYNKINRATTKGRYKCETCGKVFKSYQALGGHRASHKKNRVSNNKTEQRSETEYDNVVVVAKRIHECPICLRVFASGQALGGHKRSHGVGNLSVNQQRRVHRNESVKQRMIDLNLPAPTEEDEVSVVFQ

| **Table S2. Primers used in this study** | | |
| --- | --- | --- |
| Primers for quantative RT-PCR in apple | | |
| Gene name | Forward | Reverse |
| MdCHS | GGAGACAACTGGAGAAGGACTGGAA | CGACATTGATACTGGTGTCTTC |
| MdCHI | GGGATAACCTCGCGGCCAAA | GCATCCATGCCGGAAGCTACAA |
| MdF3H | TGGAAGCTTGTGAGGACTGGGGT | CTCCTCCGATGGCAAATCAAAGA |
| MdDFR | GATAGGGTTTGAGTTCAAGTA | TCTCCTCAGCAGCCTCAGTTTTCT |
| MdANS | CCAAGTGAAGCGGGTTGTGCT | CAAAGCAGGCGGACAGGAGTAGC |
| MdUFGT | CCACCGCCCTTCCAAACACTCT | CACCCTTATGTTACGCGGCATGT |
| MdGST | AGTTGTAGAAGATGGTGACT | CAGGTCGTTGAAGTTGTG |
| MdMYB1 | TGCCTGGACTCGAGAGGAAGACA | CCTGTTTCCCAAAAGCCTGTGAA |
| MdMYB114 | ATTAAGATGGTTGAACTATC | GAAGCCTAATCGTAAGAT |
| MdbHLH3 | ACCACCTCAGCCAGAACCT | CCTTCACCTTGGCTCTTAGTT |
| MdbHLH33 | TCCGACAGAAGACTCCATGATG | CGTGTTTAGCAAAAGAGTGAGCC |
| MdZAT1 | CTCAATCCAATCTTCAGT | TCTCCTTCTCATACTAACT |
| MdActin | TGACCGAATGAGCAAGGAAATTACT | TACTCAGCTTTGGCAATCCACATC |
| Primers for subcellular localization | | |
| Gene name | Forward | Reverse |
| MdZAT1-pCAMBIA2300 | AGAACACGGGGGACTCTAGAATGCCTAAGGATAGGAGGGG | CCCTTGCTCACCATCCCGGGTCACGGCTGGTTGTTGTCCG |
| Primers for transgene | | |
| Gene name | Forward | Reverse |
| MdZAT1-pRI101 | GTCGACATGCCTAAGGATAGGAGGGG | GGATCCCGGCTGGTTGTTGTCC |
| Primers for Y1H | | |
| Gene name | Forward | Reverse |
| MdZAT1-pGADT7 | GGATCCATGCCTAAGGATAGGAGGGG | GAGCTCTCACGGCTGGTTGTTGTC |
| MdCHI-pHIS2 | GAATTCTTCCGCTGCGGTGG | GAGCTCTATAGCATTAATTAATGGGTGGGA |
| MdCHS-pHIS2 | GAATTCGAACCCAGAACGCATGAGT | GAGCTCGAGATACAACTGCGAGCGAC |
| MdF3H-pHIS2 | GAATTCTGTGTCCGTTTATTGTGCAGTT | GAGCTCGGGAGAGACCCGCTTCTC |
| MdDFR-pHIS2 | GAATTCCTGTTCGTTTGTAATGGCAT | GAGCTCATCTTGTGTGTATGTGCTTACC |
| MdANS-pHIS2 | GAATTCGGCTAGTTCTGTATTATGGTTGATAT | GAGCTCTAGTTGGTTCCAATTGGGTT |
| MdUFGT-pHIS2 | GAATTCGAATTCCTGAGTCCCCTGTAC | GAGCTCGAGCTCACTGGAGTGGACA |
| MdGST-pHIS2 | GAATTCGAAACTGTCGTTCGTTGAAATTTAGCT | GAGCTCCTTCTTGTACAACAAAGTGC |
| MdbHLH3-pHIS2 | GAATTCATTGAAAATTTAATTAAAACTTAACTAAAACTCT | GAGCTCGTTAGTTGCATCCCAAGAAATACC |
| MdbHLH33-pHIS2 | GAATTCTAACAGTCAAACTTAACATAAAATTTAACG | GAGCTCGGTGTGGGAGTTCTGCCT |
| MdMYB1-pHIS2 | GAATTCTATAAAATATCGATGATATCGGAAATATC | GAGCTCGCTCCCCTTCCACGC |
| MdMYB114-pHIS2 | ACTCACTATAGGGCGAATTCCCGATATTATCGATATTTTGGTCCATGG | ATTCGCGAACGCGTGAGCTCTCTCTTATCTGCCTGCTAGCCAC |
| Primers for LUC assay | | |
| Gene name | Forward | Reverse |
| MdZAT1-pGreenⅡ62-SK | GCTCTAGAACTAGTGGATCCATGCCTAAGGATAGGAGGGG | TCGACGGTATCGATAAGCTTTCACGGCTGGTTGTTGTCCG |
| MdCHI-pGreenⅡ0800 | AAGCTTTTCCGCTGCGGTGG | AAGTCCTATAGCATTAATTAATGGGTGGGAC |
| MdANS-pGreenⅡ0800 | TCGACGGTATCGATAAGCTTGGCTAGTTCTGTATTATGGT | GCTCTAGAACTAGTGGATCCTAGTTGGTTCCAATTGGGTT |
| MdMYB114-pGreenⅡ0800 | TCGACGGTATCGATAAGCTTCCGATATTATCGATATTTTG | GCTCTAGAACTAGTGGATCCTCTCTTATCTGCCTGCTAGC |
| Primers for ChIP assay | | |
|  | Forward | Reverse |
| M1 | ATCTCTAGTTGAAGGAAGTAGTCCA | TATAAAAAATTAATATAAAATGTTAACGTGATT |
| M2 | TTAACATAACTTAACCGTGATCGT | TAGCATGTGATCGGCACA |
| C1 | GAGTAAGTATCCCTAATTGATTTAAATTT | AATTCTAGAGACGACACGTGAAT |
| C2 | TGAGTTGCAGAGTTCCACAC | TTAATGGTGTACTCTTATGTCATGAA |
| A1 | TCCGTCAAAGTTTCGTCAA | TTTGTTAGAACTTACTCAAAATGAGTC |
| A2 | CATGAATCCCAGATATGTTGAC | ATAGTAGTTTTAACTCAATTCGAGAAACT |
| A3 | GCATTTACTAAGGCGGTTAGG | TAGTTGGTTCCAATTGGGTT |
| Primers for VIGS | | |
| Gene name | Forward | Reverse |
| MdZAT1-pTRV2 | GGATCCTACCAGACCAGCTG | CTCGAGCTGATCCGAAGC |
| Probes for EMSA assay | | |
| Gene name | Forward | Reverse |
| MdCHI-probe | TATTAAAGATAGGATTTGGGTAATTAATCATTATCT | AGATAATGATTAATTACCCAAATCCTATCTTTAATA |
| MdANS-probe | GGATTATTGCTCCAATTGGGTTAAAGTTGTGGGACC | GGTCCCACAACTTTAACCCAATTGGAGCAATAATCC |
|  | CTAAATTTTGTAAACCCAATTGGAACCAACTA | TAGTTGGTTCCAATTGGGTTTACAAAATTTAG |
| MdMYB114-probe | TGTCTTTGAGGCAGATTGGGTGCCCTTCCCACTCCC | GGGAGTGGGAAGGGCACCCAATCTGCCTCAAAGACA |
|  | CAGACGGTAGCTTCAACCCAAAAAAAAGGTGACAGT | ACTGTCACCTTTTTTTTGGGTTGAAGCTACCGTCTG |

| **Table S3. List of interacting proteins identified by Y1H screening.** | |
| --- | --- |
| **Y1H screening results** | |
| XM_008379726.3 | PREDICTED: Malus domestica basic leucine zipper 4-like (LOC103441023), mRNA |
| XM_008339845.3 | PREDICTED: Malus domestica GTPase ERA-like, chloroplastic (LOC103401136), mRNA |
| XM_008367639.3 | PREDICTED: Malus domestica uncharacterized LOC103429487 (LOC103429487), mRNA |
| XM_029095404.1 | PREDICTED: Malus domestica methyl-CpG-binding domain-containing protein 2-like (LOC114821928), mRNA |
| XM_017323260.2 | PREDICTED: Malus domestica protein root UVB sensitive 6-like (LOC103402459), mRNA |
| XM_029101232.1 | PREDICTED: Malus domestica mitochondrial uncoupling protein 1-like (LOC114824403), mRNA |
| XM_008377216.3 | PREDICTED: Malus domestica trihelix transcription factor GTL1 (LOC103438676), transcript variant X1, mRNA |
| XR_003776701.1 | PREDICTED: Malus domestica tubulin alpha-4 chain-like (LOC114827605), misc_RNA |
| XM_008355158.3 | PREDICTED: Malus domestica prohibitin-3, mitochondrial (LOC103416947), mRNA |
| XM_029105995.1 | PREDICTED: Malus domestica quinone oxidoreductase-like protein 2 homolog (LOC114826140), mRNA |
| XM_008393532.3 | PREDICTED: Malus domestica fructose-bisphosphate aldolase 1, cytoplasmic (LOC103453937), mRNA |
| XM_008366099.3 | PREDICTED: Malus domestica succinate--CoA ligase [ADP-forming] subunit beta, mitochondrial (LOC103428008), mRNA |
| XM_008345135.3 | PREDICTED: Malus domestica chitinase-like protein 1 (LOC103406122), mRNA |
| XM_008349385.3 | PREDICTED: Malus domestica 3-oxo-Delta(4,5)-steroid 5-beta-reductase-like (LOC103410710), mRNA |
| XM_029097019.1 | PREDICTED: Malus domestica trafficking protein particle complex subunit 12 (LOC103405587), mRNA |
| XM_008371816.3 | PREDICTED: Malus domestica uncharacterized LOC103433552 (LOC103433552), mRNA |
| XM_029094030.1 | PREDICTED: Malus domestica BRASSINOSTEROID INSENSITIVE 1-associated receptor kinase 1-like (LOC103416332), transcript variant X27, mRNA |
| XM_029091424.1 | PREDICTED: Malus domestica uncharacterized LOC103414332 (LOC103414332), transcript variant X1, mRNA |
| XM_029104989.1 | PREDICTED: Malus domestica callose synthase 2-like (LOC103438357), mRNA |
| XM_029088713.1 | PREDICTED: Malus domestica COP9 signalosome complex subunit 2 (LOC103427255), mRNA |
| XM_017323009.2 | PREDICTED: Malus domestica NADH-cytochrome b5 reductase-like protein (LOC103400532), mRNA |
| XM_008348676.3 | PREDICTED: Malus domestica E3 ubiquitin-protein ligase RING1-like (LOC103409886), transcript variant X2, mRNA |
| XM_029104614.1 | PREDICTED: Malus domestica geraniol 8-hydroxylase-like (LOC103428208), transcript variant X2, mRNA |
| XM_029101395.1 | PREDICTED: Malus domestica pyrrolidone-carboxylate peptidase 1 (LOC103434061), transcript variant X1, mRNA |
| XM_008357539.3 | PREDICTED: Malus domestica copper transporter 6-like (LOC103419433), mRNA |
| XM_008385182.3 | PREDICTED: Malus domestica ferredoxin--NADP reductase, root isozyme, chloroplastic-like (LOC103446115), mRNA |
| XM_008388720.3 | PREDICTED: Malus domestica fructose-bisphosphate aldolase 1, cytoplasmic-like (LOC103449399), mRNA |
| XM_008374779.3 | PREDICTED: Malus domestica annexin D5-like (LOC103436359), mRNA |
| XM_008379882.3 | PREDICTED: Malus domestica aspartate aminotransferase, cytoplasmic (LOC103441178), mRNA |
| XM_008392859.3 | PREDICTED: Malus domestica uncharacterized LOC103453320 (LOC103453320), mRNA |
| XR_526281.3 | PREDICTED: Malus domestica uncharacterized LOC103415782 (LOC103415782), ncRNA |
| XM_008350206.3 | PREDICTED: Malus domestica ER lumen protein-retaining receptor (LOC103411579), mRNA |
| XM_008342375.3 | PREDICTED: Malus domestica huntingtin-interacting protein K-like (LOC103403535), mRNA |
| XM_008388720.3 | PREDICTED: Malus domestica fructose-bisphosphate aldolase 1, cytoplasmic-like (LOC103449399), mRNA |
| XM_008366600.3 | PREDICTED: Malus domestica 21 kDa protein-like (LOC103428499), mRNA |
| XM_008361395.3 | PREDICTED: Malus domestica heavy metal-associated isoprenylated plant protein 3-like (LOC103423320), mRNA |
| XM_008395182.3 | PREDICTED: Malus domestica guanine nucleotide-binding protein subunit beta-like protein (LOC103455608), mRNA |
| XM_008341155.3 | PREDICTED: Malus domestica uncharacterized protein At5g49945-like (LOC103402409), mRNA |
| XM_029108872.1 | PREDICTED: Malus domestica charged multivesicular body protein 7 (LOC103444482), mRNA |
| XM_008349424.3 | PREDICTED: Malus domestica probable sarcosine oxidase (LOC103410757), mRNA |
| XM_029109600.1 | PREDICTED: Malus domestica 14-3-3-like protein 16R (LOC114827559), mRNA |
| XM_029089006.1 | PREDICTED: Malus domestica protochlorophyllide reductase, chloroplastic-like (LOC103448652), mRNA |
| XM_029090288.1 | PREDICTED: Malus domestica uncharacterized LOC103449373 (LOC103449373), mRNA |
| XM_008340537.3 | PREDICTED: Malus domestica extradiol ring-cleavage dioxygenase-like (LOC103401823), mRNA |
| XM_017336619.2 | PREDICTED: Malus domestica GATA transcription factor 5-like (GATA7), transcript variant X1, mRNA |
| XM_008343268.3 | PREDICTED: Malus domestica peroxidase 63-like (LOC103404368), mRNA |
| XM_029094285.1 | PREDICTED: Malus domestica lysine-rich arabinogalactan protein 18 (LOC114821574), mRNA |
| XM_008355635.2 | PREDICTED: Malus domestica glutamine synthetase cytosolic isozyme (LOC103417448), mRNA |
| XM_050272673.1 | PREDICTED: Malus sylvestris uncharacterized LOC126605302 (LOC126605302), mRNA |
| XM_008393076.3 | PREDICTED: Malus domestica anoctamin-like protein At1g73020 (LOC103453540), transcript variant X2, mRNA |
| XM_008343022.3 | PREDICTED: Malus domestica mitochondrial phosphate carrier protein 3, mitochondrial-like (LOC103404139), mRNA |
| XM_029095639.1 | PREDICTED: Malus domestica AT-hook motif nuclear-localized protein 13-like (LOC103417163), mRNA |
| XM_029107361.1 | PREDICTED: Malus domestica 11S globulin seed storage protein 2-like (LOC114826646), mRNA |
| XM_029103369.1 | PREDICTED: Malus domestica protein PYRICULARIA ORYZAE RESISTANCE 21 (LOC103434405), mRNA |
| XM_017331554.2 | PREDICTED: Malus domestica universal stress protein PHOS34-like (LOC103432586), transcript variant X2, mRNA |
| XM_017330586.2 | PREDICTED: Malus domestica xylose isomerase-like (LOC103429066), transcript variant X2, mRNA |
| XM_008340415.3 | PREDICTED: Malus domestica probable ATP synthase 24 kDa subunit, mitochondrial (LOC103401698), mRNA |
| XM_008364425.3 | PREDICTED: Malus domestica E3 ubiquitin-protein ligase SINAT3-like (LOC103426330), transcript variant X2, mRNA |
| XM_008373374.3 | PREDICTED: Malus domestica 60S ribosomal protein L18a-2 (LOC103434990), transcript variant X1, mRNA |
| XM_029093779.1 | PREDICTED: Malus domestica phosphatidylinositol transfer protein 3-like (LOC114821490), mRNA |
| XM_008363888.3 | PREDICTED: Malus domestica triacylglycerol lipase 2-like (LOC103425788), mRNA |
| XM_008357628.3 | PREDICTED: Malus domestica GDT1-like protein 4 (LOC103419528), mRNA |
| XM_008341945.3 | PREDICTED: Malus domestica glyceraldehyde-3-phosphate dehydrogenase, cytosolic (LOC103403121), mRNA |
| XM_029094320.1 | C2H2-type zinc finger protein involved in salt tolerance. Induced by salt stress. |
| XM_008355158.3 | PREDICTED: Malus domestica prohibitin-3, mitochondrial (LOC103416947), mRNA |
| XM_008372858.3 | PREDICTED: Malus domestica lactoylglutathione lyase GLX1 (LOC103434492), mRNA |
| XM_029109937.1 | PREDICTED: Malus domestica probable mitochondrial import inner membrane translocase subunit TIM21 (LOC103412184), transcript variant X1, mRNA |
| XM_008373699.3 | PREDICTED: Malus domestica zinc transporter 1-like (LOC103435313), mRNA |
